# Association between low density lipoprotein cholesterol and all-cause mortality: results from the NHANES 1999–2014

**DOI:** 10.1038/s41598-021-01738-w

**Published:** 2021-11-11

**Authors:** Ya Liu, Fubin Liu, Liwen Zhang, Junxian Li, Wenjuan Kang, Mingli Cao, Fangfang Song, Fengju Song

**Affiliations:** 1 Department of Epidemiology and Biostatistics, National Clinical Research Center for Cancer, Key Laboratory of Cancer Prevention and Therapy of Tianjin, Tianjin’s Clinical Research Center for Cancer, Key Laboratory of Molecular Cancer Epidemiology of Tianjin, Key Laboratory of Breast Cancer Prevention and Therapy in Ministry of Education, Tianjin Medical University Cancer Institute and Hospital, Huanhu Xi Road, Tiyuan Bei, Hexi District, Tianjin, 300060 People’s Republic of China; 2 Department of Chronic Disease, Hexi Center for Disease Control, Tianjin, 300211 People’s Republic of China

**Keywords:** Health care, Medical research

## Abstract

The association between low density lipoprotein cholesterol (LDL-C) and all-cause mortality has been examined in many studies. However, inconsistent results and limitations still exist. We used the 1999–2014 National Health and Nutrition Examination Survey (NHANES) data with 19,034 people to assess the association between LDL-C level and all-cause mortality. All participants were followed up until 2015 except those younger than 18 years old, after excluding those who died within three years of follow-up, a total of 1619 deaths among 19,034 people were included in the analysis. In the age-adjusted model (model 1), it was found that the lowest LDL-C group had a higher risk of all-cause mortality (HR 1.708 [1.432–2.037]) than LDL-C 100–129 mg/dL as a reference group. The crude-adjusted model (model 2) suggests that people with the lowest level of LDL-C had 1.600 (95% CI [1.325–1.932]) times the odds compared with the reference group, after adjusting for age, sex, race, marital status, education level, smoking status, body mass index (BMI). In the fully-adjusted model (model 3), people with the lowest level of LDL-C had 1.373 (95% CI [1.130–1.668]) times the odds compared with the reference group, after additionally adjusting for hypertension, diabetes, cardiovascular disease, cancer based on model 2. The results from restricted cubic spine (RCS) curve showed that when the LDL-C concentration (130 mg/dL) was used as the reference, there is a U-shaped relationship between LDL-C level and all-cause mortality. In conclusion, we found that low level of LDL-C is associated with higher risk of all-cause mortality. The observed association persisted after adjusting for potential confounders. Further studies are warranted to determine the causal relationship between LDL-C level and all-cause mortality.

## Introduction

For decades, the high level of low-density lipoprotein cholesterol (LDL-C) has been considered as the main cause of the development and death of atherosclerotic cardiovascular diseases^1^. A large number of studies have strongly shown that the reduction of plasma LDL-C concentration by lipid-lowering drugs is related to the greater reduction of the development and mortality of cardiovascular diseases^2–9^.

However, many studies have found the opposite result to previous studies on cardiovascular diseases, and LDL-C level is negatively correlated with all-cause mortality of patients^10,11^.

In the relatively healthy population, the correlation between low LDL-C concentration and mortality is still uncertain. Observational studies have shown that the risk of infectious diseases and cancer in healthy individuals with low LDL-C concentration is significantly increased^12,13^. These studies have raised an important question, that is, whether the low LDL-C level is related to the all-cause mortality and cancer mortality of in the general population. In some randomized controlled trials or observational studies, subjects with abnormally low LDL-C concentration were excluded from the analysis. This suggests that the relationship between low LDL-C level and mortality in the general population needs to be further explored.

A mouse experimental study pointed out that LDL-C receptor can regulate the expression of sensory neurons, while sensory impairments can increase the risk of all-cause mortality^14^. Therefore, we conduct a study, in which we aim to further explore the potential relationship between LDL-C level and all-cause mortality according to the horizontal data of NHANES study. Because the main goal of disease prevention is to prolong life, all-cause mortality is the most important and easy to determine result, and there is the smallest risk of deviation among all outcome indicators, so we mainly choose to focus on the relationship between LDL-C level and all-cause mortality. In addition, we reveal the relationship between LDL-C level and cardiovascular mortality as a secondary analysis result.

## Results

### Baseline characteristics by different LDL-C levels of the study population

A total of 19,034 participants (mean age 46.44 years) were recruited in our study, including 9045 males and 9989 females. After a median follow-up of 7.83 years, excluding those who died within three years of follow-up, a total of 1619 deaths among 19,034 participants were included in the analysis. Five groups were defined according to the level of baseline LDL-C concentration (< 70, 70–99, 100–129, 130–159, ≥ 160 mg/dL). Table 1 represents baseline characteristics of study participants respectively according to different levels of LDL-C concentrations at baseline. Under different LDL-C classification levels, all social demographic and health-related disease factors have statistical significance except whether cancer exists at baseline.

**Table 1:** Baseline characteristics of 19,034 individuals in the NHANES Study, 1999–2014.

Characteristics	All categories, N = 19,034	LDL-C (mg/dL)					*P*_value_
		< 70, N = 1557 N =	70–99, N = 5246 N =	100–129, N = 6212 N =	130–159, N = 3996 N =	≥ 160, N = 2023 N =	
Age (years)^a^	46.4(19.1)	43.8(22.1)	43.1(20.3)	46.8(18.6)	49.2(17.3)	50.6(16.5)	< 0.001
BMI (kg/m^2^)^a^	27.3(7.2)	26.4(7.6)	26.8(7.0)	27.5(7.5)	27.9(7.0)	27.9(6.8)	< 0.001
**Sex**^b^							
Men	9045(47.5)	781(50.2)	2432(46.4)	2917(47.0)	1956(48.9)	959(47.4)	0.024
Women	9989(52.5)	776(49.8)	2814(53.6)	3295(53.0)	2040(51.1)	1064(52.6)	
**Race**^b^							
Non-Hispanic White	8656(45.5)	663(42.6)	2309(44.0)	2866(46.2)	1851(46.3)	967(47.8)	< 0.001
Non-Hispanic Black	3872(20.3)	398(25.5)	1142(21.8)	1200(19.3)	738(18.5)	394(19.5)	
Mexican American	3674(19.3)	266(17.1)	997(19.0)	1232(19.8)	813(20.3)	366(18.1)	
Other Hispanic	1454(7.7)	101(6.5)	373(7.1)	471(7.6)	343(8.6)	166(8.2)	
Other Race	1378(7.2)	129(8.3)	425(8.1)	443(7.1)	251(6.3)	130(6.4)	
**Marital status**^b^							
Single	4414(23.2)	492(34.2)	1511(30.5)	1413(23.5)	694(17.7)	304(15.4)	< 0.001
Married	10,228(53.7)	684(47.5)	2541(51.4)	3405(56.7)	2399(62.2)	1199(60.8)	
Separated/divorced/widowed	3643(19.1)	263(18.3)	894(18.1)	1191(19.8)	826(21.1)	469(23.8)	
**Education level**^b^							
Primary school	2156(11.3)	149(11.3)	519(11.4)	690(11.9)	513(13.3)	285(14.4)	< 0.001
Junior high school	2710(14.3)	218(16.6)	703(15.5)	900(15.5)	573(14.8)	316(16.0)	
Senior high school	3993(21.0)	300(22.8)	990(21.8)	1332(23.0)	875(22.7)	496(25.1)	
College and above	8639(45.4)	646(49.2)	2334(51.3)	2879 (49.6)	1900(49.2)	880(44.5)	
**Smoking status**^b^							
Never smoker	9623(56.2)	711(54.4)	2547(56.9)	3182(56.1)	2158(57.5)	1025(53.3)	0.003
Former smoker	4345(25.4)	339(26.0)	1072(24.0)	1498(26.5)	933(24.8)	503(26.2)	
Current smoker	3157(18.4)	256(19.6)	856(19.1)	987(17.4)	663(17.7)	395(20.5)	
**Hypertension**^b^							
No	13,015(68.7)	1003(64.7)	3619(69.4)	4291(69.5)	2718(68.3)	1384(68.8)	0.005
Yes	5924(31.3)	548(35.3)	1599(30.6)	1887(30.5)	1261(31.7)	629(31.2)	
**Diabetes**^b^							
No	17,139(90.1)	1258(80.8)	4632(88.3)	5682(91.5)	3691(92.4)	1876(92.7)	< 0.001
Yes	1889 (9.9)	299(19.2)	611(11.7)	528(8.5)	304(7.6)	147(7.3)	
**Cardiovascular Disease**^b^							
No	16,129(92.3)	1084(82.8)	4091(90.0)	5432(93.8)	3655(95.0)	1867(94.4)	< 0.001
Yes	1338(7.7)	225(17.2)	454(10.0)	358(6.2)	191(5.0)	110(5.6)	
**Cancer**^b^							
No	16,027(91.5)	1187(90.5)	4152(91.2)	5301(91.3)	3556(92.1)	1831(92.4)	0.147
Yes	1484(8.5)	125(9.5)	403(8.8)	502(8.7)	304(7.9)	150(7.6)	

*BMI* Body mass index, *LDL-C* low-density lipoprotein cholesterol.

^a^Mean (Standard Deviation), *p* values from ANOVA.

^b^N (%), *p* values from the χ2 test.

### Association between LDL-C level and all-cause mortality

**Figure 1:**
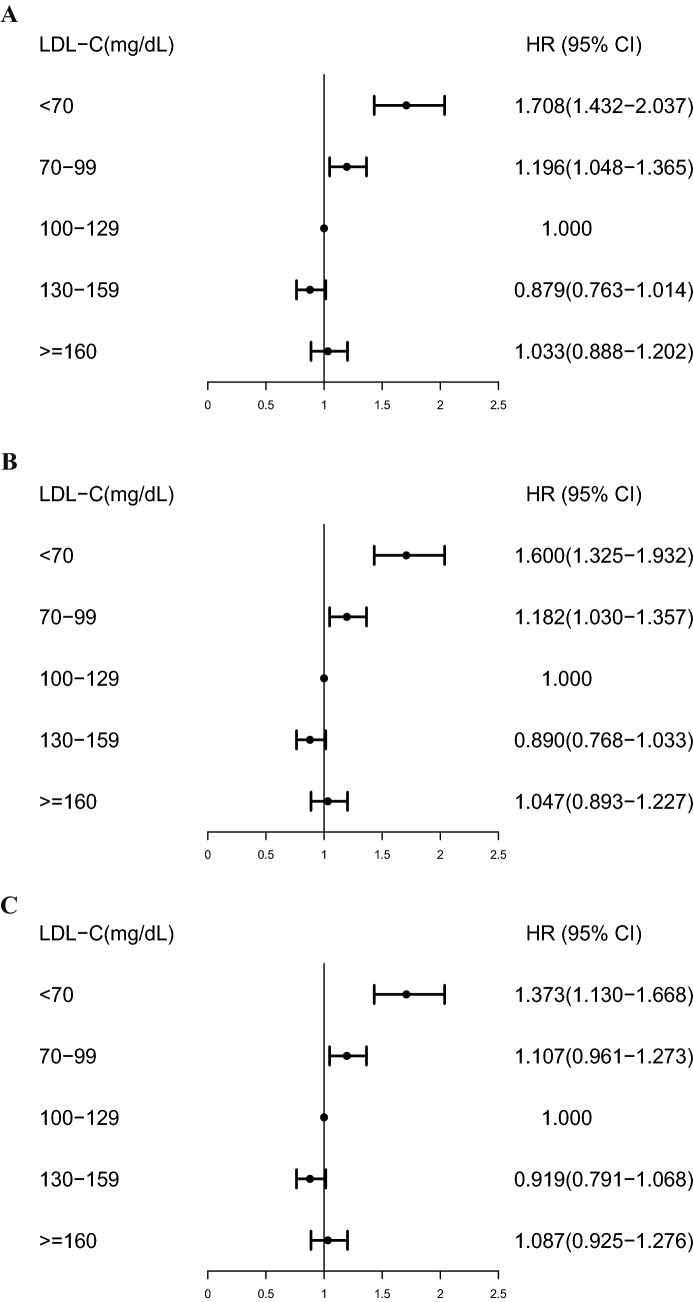
Multivariate analysis of low-density lipoprotein cholesterol (LDL-C) level and covariates associated with all-cause mortality in the NHANES Study, 1999–2014. (**A**) Age-adjusted model (model 1), adjusted for age (continuous). (**B**) Crude-adjusted model (model 2), adjusted for age (continuous), sex, race, marital status, education level, smoking status, BMI (continuous). (**C**) Fully-adjusted model (model 3), adjusted for age (continuous), sex, race, marital status, education level, smoking status, BMI (continuous), hypertension, diabetes, cardiovascular disease, cancer.

Table 2 makes a univariate analysis on the relationship between covariates including LDL-C level and all-cause mortality. The results show that the second lowest quintile of LDL-C has the lowest risk of all-cause mortality and show that other covariates have statistical significance for all-cause mortality. We provided three models: age-adjusted (model 1), crude-adjusted (model 2) and fully-adjusted (model 3) model. HR from the cox proportional hazards regression models was assessed the associations between LDL-C level and all-cause mortality (Fig. 1, Table S1). In the age-adjusted model (model 1), it was found that the lowest LDL-C level had a higher risk of all-cause mortality (HR 1.708 [1.432–2.037]) than LDL-C (100–129 mg/dL) as a reference group. After adjusting for age, sex, race, marital status, education level, smoking status and BMI, crude-adjusted model (model 2) also observed a higher risk of all-cause mortality in the lowest LDL-C level (HR 1.600 [1.325–1.932]). The fully-adjusted model (model 3) additionally adjusted for hypertension, diabetes, cardiovascular disease and cancer based on model 2, and the relationship between the lowest LDL-C level and the higher risk of all-cause mortality was still significant (HR 1.373 [1.130–1.668]).

**Table 2:** Univariate analysis of LDL-C level and covariates associated with all-cause mortality in the NHANES Study, 1999–2014.

Characteristics	Events/numbers	HR (95% CI)	*P*
**LDL-C (mg/dL)**			
< 70	161/1557	1.521 (1.276–1.812)	< 0.001
70–99	398/5246	0.992 (0.870–1.132)	0.908
100–129	504/6212	1 (ref)	
130–159	352/3996	0.993 (0.861–1.145)	0.925
≥ 160	204/2023	1.218 (1.047–1.416)	0.011
**Sex**			
Men	883/9045	1 (ref)	
Women	736/9989	0.725 (0.658–0.800)	< 0.001
**Race**			
Non-Hispanic White	964/8656	1 (ref)	
Non-Hispanic Black	260/3872	0.624 (0.544–0.716)	< 0.001
Mexican American	292/3674	0.602 (0.528–0.686)	< 0.001
Other Hispanic	62/1454	0.513 (0.397–0.664)	< 0.001
Other Race	41/1378	0.463 (0.339–0.633)	< 0.001
**Marital status**			
Single	126/4414	1 (ref)	
Married	854/10,228	2.834 (2.351–3.417)	< 0.001
Separated/divorced/widowed	605/3643	6.667 (5.502–8.079)	< 0.001
**Education level**			
Primary school	388/2156	1 (ref)	
Junior high school	276/2710	0.589 (0.505–0.687)	< 0.001
Senior high school	384/3993	0.566 (0.491–0.652)	< 0.001
College and above	543/8639	0.393 (0.345–0.448)	< 0.001
**Smoking status**			
Never smoker	663/9623	1 (ref)	
Former smoker	598/4345	1.941 (1.738–2.168)	< 0.001
Current smoker	290/3157	1.250 (1.089–1.436)	0.002
**Hypertension**			
No	770/13,015	1 (ref)	
Yes	846/5924	2.953 (2.678–3.257)	< 0.001
**Diabetes**			
No	1286/17,139	1 (ref)	
Yes	330/1889	2.976 (2.636–3.360)	< 0.001
**Cardiovascular Disease**			
No	1215/16,129	1 (ref)	
Yes	366/1338	4.436 (3.945–4.987)	< 0.001
**Cancer**			
No	1287/16,027	1 (ref)	
Yes	309/1484	3.128 (2.762–3.543)	< 0.001

*CIs* Confidence intervals, *HR* hazard ratio, *LDL-C* low-density lipoprotein cholesterol.

**Figure 2:**
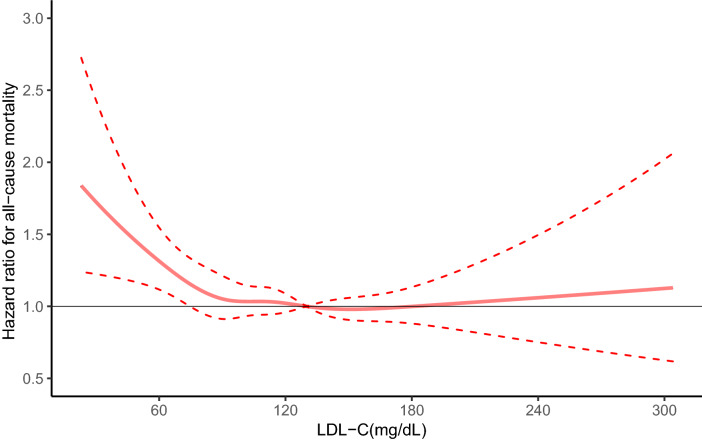
Spline plot of low-density lipoprotein cholesterol (LDL-C) level and all-cause mortality rate. The adjusted odd ratios and 95% confidence intervals (CIs) were calculated with logistic regression models after adjusting for age (continuous), sex, race, marital status, education level, smoking status, BMI (continuous), hypertension, diabetes, cardiovascular disease, cancer.

Moreover, the RCS curve (Fig. 2) showed that when the LDL-C concentration (130 mg/dL) was used as the reference, a lower LDL-C concentration was associated with a higher all-cause mortality risk. And there is a U-shaped relationship between LDL-C level and all-cause mortality after adjusting for a series of potential confounders.

Stratified analysis of the relationship between LDL-C and all-cause mortality was shown in Table 3. We found that there was a significant difference between the lowest LDL-C level and the higher risk of all-cause mortality among people who were Non-Hispanic White, Mexican American, other Hispanic, married, junior high school educated, senior high school educated, former smoker, current smoker, with a history of cardiovascular disease, without a history of cancer and with or without history of hypertension and diabetes.

**Table 3:** Stratified analysis of the relationship between LDL-C level and all-cause mortality in the NHANES Study, 1999–2014.

Characteristics	LDL-C (mg/dL)	Numbers of Events	HR (95% CI)	*P*
**Sex**				
Men	< 70	101	1.315 (1.023–1.690)	0.033
	70–99	225	1.071 (0.887–1.294)	0.475
	100–129	284	1 (ref)	
	130–159	182	0.898 (0.733–1.100)	0.300
	≥ 160	91	1.042 (0.828–1.312)	0.724
Women	< 70	60	1.460 (1.068–1.998)	0.018
	70–99	173	1.133 (0.916–1.401)	0.251
	100–129	220	1 (ref)	
	130–159	170	0.946 (0.756–1.183)	0.627
	≥ 160	113	1.125 (0.896–1.413)	0.311
**Race**				
Non-Hispanic White	< 70	94	1.318 (1.024–1.696)	0.032
	70–99	234	1.080 (0.902–1.293)	0.404
	100–129	305	1 (ref)	
	130–159	219	0.935 (0.774–1.130)	0.487
	≥ 160	112	1.060 (0.860–1.306)	0.586
Non-Hispanic Black	< 70	28	1.441 (0.891–2.329)	0.136
	70–99	69	1.059 (0.740–1.514)	0.755
	100–129	77	1 (ref)	
	130–159	44	0.913 (0.609–1.369)	0.659
	≥ 160	42	1.066 (0.718–1.581)	0.752
Mexican American	< 70	27	1.662 (1.022–2.703)	0.040
	70–99	71	1.246 (0.876–1.773)	0.221
	100–129	87	1 (ref)	
	130–159	68	0.915 (0.634–1.319)	0.633
	≥ 160	39	1.525 (1.039–2.237)	0.031
Other Hispanic	< 70	8	3.744 (1.484–9.443)	0.005
	70–99	16	1.690 (0.805–3.549)	0.165
	100–129	20	1 (ref)	
	130–159	13	0.827 (0.358–1.910)	0.656
	≥ 160	5	0.563 (0.208–1.522)	0.257
Other Race	< 70	4	0.497 (0.129–1.914)	0.310
	70–99	8	0.702 (0.278–1.774)	0.454
	100–129	15	1 (ref)	
	130–159	8	0.766 (0.260–2.252)	0.628
	≥ 160	6	1.382 (0.496–3.846)	0.536
**Marital status**				
Single	< 70	15	1.280 (0.604–2.713)	0.520
	70–99	38	1.101 (0.661–1.832)	0.712
	100–129	46	1 (ref)	
	130–159	16	0.508 (0.261–0.988)	0.046
	≥ 160	11	0.875 (0.443–1.728)	0.700
Married	< 70	87	1.475 (1.142–1.906)	0.003
	70–99	206	1.136 (0.936–1.378)	0.196
	100–129	256	1 (ref)	
	130–159	204	1.024 (0.840–1.250)	0.812
	≥ 160	101	1.286 (1.032–1.604)	0.025
Separated/divorced/widowed	< 70	57	1.237 (0.889–1.723)	0.207
	70–99	148	1.081 (0.861–1.357)	0.504
	100–129	227	1 (ref)	
	130–159	147	0.848 (0.662–1.087)	0.194
	≥ 160	103	0.916 (0.712–1.179)	0.495
**Education level**				
Primary school	< 70	29	1.203 (0.770–1.879)	0.418
	70–99	109	1.259 (0.947–1.675)	0.113
	100–129	121	1 (ref)	
	130–159	74	0.785 (0.564–1.091)	0.150
	≥ 160	55	0.904 (0.651–1.254)	0.545
Junior high school	< 70	36	1.712 (1.107–2.647)	0.016
	70–99	62	1.218 (0.861–1.724)	0.265
	100–129	83	1 (ref)	
	130–159	59	0.981 (0.680–1.415)	0.918
	≥ 160	36	1.336 (0.911–1.959)	0.139
Senior high school	< 70	34	1.562 (1.031–2.367)	0.035
	70–99	97	1.271 (0.947–1.705)	0.110
	100–129	108	1 (ref)	
	130–159	94	1.193 (0.874–1.629)	0.267
	≥ 160	51	1.448 (1.053–1.990)	0.023
College and above	< 70	58	1.296 (0.939–1.788)	0.114
	70–99	119	0.900 (0.708–1.143)	0.388
	100–129	184	1 (ref)	
	130–159	122	0.818 (0.642–1.043)	0.105
	≥ 160	60	0.888 (0.666–1.185)	0.421
**Smoking status**				
Never smoker	< 70	57	1.227 (0.894–1.684)	0.206
	70–99	149	0.977 (0.783–1.218)	0.834
	100–129	201	1 (ref)	
	130–159	151	0.919 (0.731–1.155)	0.469
	≥ 160	105	1.188 (0.940–1.502)	0.150
Former smoker	< 70	66	1.355 (1.006–1.826)	0.045
	70–99	150	1.118 (0.893–1.400)	0.330
	100–129	197	1 (ref)	
	130–159	134	0.908 (0.714–1.155)	0.433
	≥ 160	51	0.990 (0.747–1.311)	0.942
Current smoker	< 70	25	1.720 (1.081–2.737)	0.022
	70–99	76	1.343 (0.971–1.859)	0.075
	100–129	86	1 (ref)	
	130–159	59	0.936 (0.654–1.341)	0.720
	≥ 160	44	1.029 (0.711–1.489)	0.881
**Hypertension**				
No	< 70	57	1.406 (1.015–1.946)	0.040
	70–99	165	1.038 (0.839–1.283)	0.732
	100–129	249	1 (ref)	
	130–159	187	0.826 (0.668–1.021)	0.077
	≥ 160	112	1.047 (0.841–1.304)	0.680
Yes	< 70	104	1.416 (1.107–1.813)	0.006
	70–99	232	1.184 (0.980–1.431)	0.081
	100–129	255	1 (ref)	
	130–159	163	1.026 (0.829–1.270)	0.816
	≥ 160	92	1.114 (0.878–1.414)	0.375
**Diabetes**				
No	< 70	91	1.290 (1.007–1.652)	0.044
	70–99	303	1.186 (1.013–1.389)	0.034
	100–129	413	1 (ref)	
	130–159	302	0.924 (0.786–1.087)	0.341
	≥ 160	177	1.058 (0.889–1.259)	0.527
Yes	< 70	70	1.554 (1.101–2.194)	0.012
	70–99	93	0.911 (0.669–1.241)	0.554
	100–129	90	1 (ref)	
	130–159	50	0.890 (0.598–1.323)	0.563
	≥ 160	27	1.286 (0.845–1.958)	0.241
**Cardiovascular Disease**				
No	< 70	97	1.341 (0.971–1.852)	0.075
	70–99	333	1.161 (0.956–1.410)	0.131
	100–129	455	1 (ref)	
	130–159	341	1.037 (0.862–1.248)	0.700
	≥ 160	189	1.092 (0.872–1.369)	0.443
Yes	< 70	73	1.412 (1.024–1.946)	0.035
	70–99	102	0.869 (0.650–1.161)	0.342
	100–129	102	1 (ref)	
	130–159	50	0.807 (0.560–1.162)	0.248
	≥ 160	39	1.088 (0.747–1.586)	0.660
**Cancer**				
No	< 70	122	1.394 (1.116–1.741)	0.003
	70–99	312	1.148 (0.981–1.343)	0.086
	100–129	395	1 (ref)	
	130–159	290	0.940 (0.795–1.110)	0.463
	≥ 160	168	1.077 (0.901–1.286)	0.416
Yes	< 70	35	1.391 (0.926–2.090)	0.112
	70–99	76	1.000 (0.726–1.376)	0.999
	100–129	103	1 (ref)	
	130–159	61	0.898 (0.630–1.278)	0.550
	≥ 160	34	1.243 (0.851–1.817)	0.261

Adjusted for age (continuous), sex, race, marital status, education level, smoking status, BMI (continuous), hypertension, diabetes, cardiovascular disease, cancer.

*CIs* Confidence intervals, *HR* hazard ratio, *LDL-C* low-density lipoprotein cholesterol.

### Association between LDL-C level and cardiovascular mortality

The results of univariate analysis show that the fourth level of LDL-C concentration has the lowest risk of cardiovascular mortality and show that other covariates have statistical significance for all-cause mortality (Table S2).

Similar to the results between LDL-C level and all-cause mortality, the association between LDL-C level and cardiovascular mortality of multivariate analysis indicates that the lowest level of LDL-C concentration has a higher risk of cardiovascular mortality and there is a U-shaped relationship between LDL-C level and cardiovascular mortality after adjusting for a series of potential confounders (Table S3, Supplement Fig. 1, Supplement Fig. 2).

Stratified analysis of the relationship between LDL-C and cardiovascular mortality show that there was a significant difference between the lowest LDL-C level and the higher risk of cardiovascular mortality among people who were married, college and above educated, former smoker, and had no history of diabetes at baseline (Table S4).

## Discussion

In this study, using a nationally representative sample of the US, we found that low LDL-C level (< 70 mg/dL) is associated with increased risk of all-cause mortality. The observed association persisted after adjusting for potential confounders such as age, sex, race, marital status, education level, smoking status, BMI, hypertension, diabetes, cardiovascular disease, cancer. In addition, the RCS curve revealed the U-shaped correlation between LDL-C level and all-cause mortality on a continuous scale, and the death risk of low and high LDL-C levels increased significantly. Although the public’s attention focuses on the benefit of lipid lowering, this study highlights the potential harmful effect of very low LDL-C level. We found similar results between LDL-C levels and cardiovascular mortality.

Multiple studies have shown that low LDL-C level is associated with an increased risk of all-cause mortality, that is to say, our research results are consistent with them. A study in South Korea^15^ showed that in the population with low LDL-C level, especially in men, the death result from any cause increased. And this discovery was verified in another cohort. An ongoing prospective cohort study of the general population in Denmark^16^ shows that, the association between low LDL-C level and higher risk all-cause mortality risk was strongest in age and sex adjusted model, and decreased but still existed after adjusting baseline comorbidity. At the same time, the above two studies also found that there was a U-shaped correlation between LDL-C level and the risk of death from all causes, and low and high levels of LDL-C were associated with increased risk. CHARLS, a national representative longitudinal study in China^17^, shows that middle-aged and elderly people in China with low LDL-C level have higher risk of all-cause mortality. Another Chinese longitudinal health longevity survey (CLHLS)^18^ also showed that the higher LDL-C level was negatively correlated with the 3-year all-cause mortality of the Chinese elderly.

However, some studies have investigated the relationship between LDL-C level and the risk of all-cause mortality, and found no correlation^19–21^ or reverse correlation^10,22–24^. Besides, when we do stratification analysis, for male and female, the lowest level of LDL-C concentration increased the risk of all-cause mortality. We also found that the lowest level of LDL-C increased the risk of all-cause mortality among participants who did not have cancer, hypertension and diabetes at baseline.

At present, there are probably the following explanations for the unfavourable effects of low LDL-C level. LDL-C has been suggested to play an important role in host defence against both bacterial and viral pathogens^25^. Many animal and laboratory experiments have shown that LDL could bind to and inactivate a broad range of microorganisms and their toxic products^26–28^. It has been proposed that LDL-C may have the potential to protect against cancer as many cancer types are caused by viruses^29^. Ravnskov et al.^13^reviewed nine cohort studies including more than 140 000 individuals followed for 10–30 years and found that low cholesterol was associated with cancer^13^. In addition, cholesterol-lowering experiments on rodents have led to cancer as well^30^. In agreement with these findings, individuals with familial hypercholesterolaemia have been found to possess significantly lower cancer mortality^31^. Moreover, according to the data of 37 250 patients in the international Monitoring Dialysis Outcomes database, it was recently found that LDL-C is related to reducing infectious mortality^10^. LDL-C level is related to sensory impairments, which is a major risk factor for all-cause mortality. Many studies^32–34^ have shown that sensory impairments is associated with higher risk of all-cause mortality. Compared with the participants without sensory impairments, the risk of all-cause mortality of participants with sensory impairments increased significantly. Therefore, lower LDL-C may contribute to a higher risk of death from infection, cancer and sensory impairments, which in turn results in increased all-cause mortality.

To the best of our knowledge, this is the first study to find a significant relationship between low LDL-C level and all-cause mortality in population with a broad age range using a nationally representative sample of US (NHANES 1999–2014). The NHANES data provided us with a unique opportunity to study the association between LDL-C level and all-cause mortality in a large multiethnic, nationally representative sample of the US population. In addition, we were also able to adjust for a wide range of potential confounders such as sociodemographic characteristics and health-related disease factors to assess the true association between LDL-C level and all-cause mortality in the general population. The exclusion of subjects who died within 3 years of follow-up is helpful to reduce the influence of potential reverse causality between related results and low LDL-C concentration.

There are several limitations need to be noted. First, the self-reported information of sociodemographic characteristics and health-related disease factors may introduce potential misclassification bias. However, this is likely to be a non-differential bias, which will bias the findings to null. Second, we did not conduct a stratified analysis of whether lipid-lowering treatment was performed or not. Third, we only analyzed the LDL-C level at baseline, and we cannot rule out that the results may be affected by the start or stop of lipid-lowering therapy during the follow-up period and did not observe the dynamic changes with time. Fourth, we did not combine the information about prescription medications with all-cause mortality. Furthermore, a well-designed large-scale population study is needed to establish a specific threshold of LDL-C level for future death risk. Finally, we can't deal with the problem of causality, because the design of research is observational. In theory, this problem can be studied in Mendelian random analysis, simulating nonlinear and U-shaped relations^35–37^. However, in Mendelian random analysis, the modeling of U-shaped association requires high statistical ability and a large number of genetic instruments to explain most changes in plasma concentration of LDL-C. There is no such genetic data with sufficient statistical capacity in the NHANES study. Future studies with longitudinal data on history of recurrent depressive episodes are warranted to further confirm the findings.

## Conclusion

In a nationally representative sample of US, low LDL-C level was found to be associated with higher risk of all-cause mortality after adjusting for confounding factors, such as age, sex, race, marital status, education level, smoking status, BMI, hypertension, diabetes, cardiovascular disease, cancer. There is a U-shaped relationship between LDL-C level and all-cause mortality. Compared with the middle LDL-C level, the lower LDL-C level and the higher LDL-C level, may have higher all-cause mortality. Further studies are warranted to determine the causal relationship between LDL-C level and all-cause mortality.

## Methods

The National Center for Health Statistics, which is part of the Centers for Disease Control and Prevention, provides vital and health statistics for the United States. The National Health and Nutrition Examination Survey (NHANES) is a major project of the National Center for Health Statistics, which aims to assess the health and nutrition status of American adults and children. The NHANES began in 1960s, and the stratified and multistage probability design has been used to survey about 5,000 representative samples of non-institutional American civilian population every year since 1999. It collects health information from representative samples of American population through interviews, medical examinations and laboratory tests. The survey results are used to determine the prevalence rate and risk factors of major diseases, help to formulate public health policies, design health programs and services, and expand national health knowledge. All NHANES protocols were approved by the National Center for Health Statistics’ Research Ethics Review Board, all participants signed a consent form before their participations and all research was performed in accordance with relevant guidelines/regulations. For more detailed information about NHANES data collection is published and available at https://www.cdc.gov/nchs/nhanes.htm.

### Study population

We collected the NHANES data set from 1999 to 2014, which consisted of 82,091 participants. All participants completed a self-management questionnaire, including lifestyle, sociodemographic factors, health-related diseases and so on, and followed them up until 2015 except for under the age of 18; while some participants received physical examination and provided blood samples for biochemical measurement. According to our research purpose, we included the subjects who responded to the NHANES survey and had their cholesterol levels measured into the final analysis team, while those who lacked cholesterol measurements were excluded (56,243); in addition, we excluded participants who did not follow up (6152) and those who died within three years of follow-up (662) in order to prevent reverse causality, and finally a total of 19,034 people were included.

### Exposure

The main exposure of interest was LDL-C level. Plasma cholesterol levels were measured on subjects who were examined in the morning. LDL-C is calculated from the measured values of total cholesterol, high density lipoprotein cholesterol (HDL-C) and triglyceride, in accordance with Friedwald's calculation formula: [LDL-C] = [total cholesterol] – [HDL-C] – [triglycerides/5].

### Potential confounding variables

We took age, sex, race, education level, marital status, smoking status, body mass index (BMI), hypertension, diabetes, cancer and cardiovascular disease as potential confounding variables. These potential confounders were included in this analysis based on previous literature and availability of data in the NHANES: sociodemographic factors including age (continuous), sex (men, women), race/ethnicity (non-Hispanic white, non-Hispanic black, Mexican American, Hispanic and others), education level (primary school, junior high school, senior high school, college and above, or missing), marital status (single, married, separated/divorced/widowed, or missing), smoking status(never smoker, former smoker, current smoker, or missing), BMI (continuous), and health-related disease including hypertension (no, yes), diabetes (no, yes), cardiovascular disease (no, yes), cancer (no, yes). In addition, for smoking status, participants were categorized as never smoker, former smoker, and current smoker. Former smoker were those who reported smoking at least 100 cigarettes in their lifetime but were currently nonsmokers. Current smoker were those subjects who reported smoking at least 100 cigarettes in their lifetime and were current with daily or few days of smoking. This category has previously been used in studies of depression at the National Institutes of Health.

### Endpoints

The primary outcome of our study was all-cause mortality. The causes of death included malignant tumors, cardiovascular diseases, respiratory diseases, Alzheimer's disease, diabetes, nephropathy-related diseases, accidental death and other causes. The secondary outcome was cardiovascular death, and the results were shown in the supplementary tables.

### Statistical analysis

The main purpose of this study was to determine the relationship between LDL-C level and all-cause mortality. According to the plasma LDL-C concentration at baseline, we predefined five LDL-C classification levels: < 70, 70–99, 100–129, 130–159, ≥ 160 mg/dL, to evaluate the association between LDL-C level and all-cause mortality. The LDL-C level of 100–129 mg/dL was served as the reference category.

Descriptive analyses were performed to assess the distribution in baseline characteristics at different LDL-C levels. Categorical variables were described by quantity (percentage) and compared using the Chi-square test. Continuous variables were described by mean (standard deviation) and compared using ANOVA. Restricted cubic spline (RCS) curve was used to explore and visually show the relationship between LDL-C level and all-cause mortality on a continuous scale. Univariate logistic regression was used to evaluate the relationship between confounder variables and all-cause mortality to identify potential risk factors, expressed as hazard ratio (HR) and 95% confidence intervals (CIs). The follow-up time of participants was defined as the interval from interview to date of death or to the date they were censored. Multivariate logistic regression models were used to explore the associations between LDL-C level and all-cause mortality. We constructed three models, respectively, the age-adjusted model (model 1) which only adjusts according to age, the crude-adjusted model (model 2) which adjusts according to age, sex, race, marital status, education level, smoking status, BMI, and the fully-adjusted model (model 3) which additionally adjusts hypertension, diabetes, cardiovascular disease and cancer based on model 2. Stratified analysis was carried out according to sex, race, education level, marital status, smoking status, diabetes, hypertension, cancer, cardiovascular and cardiovascular diseases, so as to determine the potential subgroup with significant correlation between LDL-C level and all-cause mortality. The association between LDL-C level and cardiovascular mortality was analyzed in the same way as LDL-C level and all-cause mortality. The reported two-tailed *P*_value_ < 0.05 was considered to be statistically significant. All statistical analysis were performed using SPSS 23.0 software and R version 3.4.3.

## Supplementary Information

Supplementary Information.

## Abbreviations

BMI: Body mass index
CIs: Confidence intervals
HDL-C: High density lipoprotein cholesterol
LDL-C: Low density lipoprotein cholesterol
NHANES: National Health and Nutrition Examination Survey
HR: Hazard ratio
RCS: Restricted cubic spine
