# Supplementary material for: Association between low density lipoprotein cholesterol and all-cause mortality: results from the NHANES 1999–2014
Source: Sci Rep. 2021 Nov 11;11:22111. doi: 10.1038/s41598-021-01738-w (PMC8586008; doi:10.1038/s41598-021-01738-w)
Supplement: Supplementary file 1 — Supplementary Information. [file 41598_2021_1738_MOESM1_ESM.docx]

**Association between low density lipoprotein cholesterol and all-cause mortality：Results from the NHANES 1999-2014**

Ya Liu^1^, Fubin Liu^1^, Liwen Zhang^1^, Junxian Li^1^, Wenjuan Kang^1^, Mingli Cao^2^, Fangfang Song^1^, Fengju Song^1#^

***Institutional Affiliations for all the authors***

Department of Epidemiology and Biostatistics, National Clinical Research Center for Cancer, Key Laboratory of Breast Cancer Prevention and Therapy in Ministry of Education, Tianjin Medical University Cancer Institute and Hospital, Tianjin 300060, People’s Republic of China

2. Department of Chronic Disease, Hexi Center for Disease Control, Tianjin 300211, People’s Republic of China

**^#^Corresponding should be given to:** Fengju Song

**Fengju Song**

**Address:** Tianjin Medical University Cancer Institute and Hospital, Huanhu Xi Road, Tiyuan Bei, Hexi District, Tianjin 300060, P.R. China.

**Tel:** 0086-022-23372231; **Fax:** 0086**-**022-23372231; **E-mail**: [songfengju@163.com](mailto:songfengju@163.com)

**Table S1**. Multivariate analysis of LDL-C level and covariates associated with all-cause mortality in the NHANES Study, 1999–2014.

| Characteristics | Events/numbers | HR (95% CI) | | |
| --- | --- | --- | --- | --- |
|  |  | Model 1^a^ | Model 2^b^ | Model 3^c^ |
| LDL-C (mg/dL) |  |  |  |  |
| <70 | 161/1557 | 1.708(1.432-2.037) | 1.600(1.325-1.932) | 1.373(1.130-1.668) |
| 70-99 | 398/5246 | 1.196(1.048-1.365) | 1.182(1.030-1.357) | 1.107(0.961-1.273) |
| 100-129 | 504/6212 | 1 (ref) | 1 (ref) | 1 (ref) |
| 130-159 | 352/3996 | 0.879(0.763-1.014) | 0.890(0.768-1.033) | 0.919(0.791-1.068) |
| ≥160 | 204/2023 | 1.033(0.888-1.202) | 1.047(0.893-1.227) | 1.087(0.925-1.276) |
| Sex |  |  |  |  |
| Men | 883/9045 |  | 1 (ref) | 1 (ref) |
| Women | 736/9989 |  | 0.650(0.580-0.727) | 0.668(0.595-0.749) |
| Race |  |  |  |  |
| Non-Hispanic White | 964/8656 |  | 1 (ref) | 1 (ref) |
| Non-Hispanic Black | 260/3872 |  | 0.952(0.818-1.108) | 0.958(0.820-1.118) |
| Mexican American | 292/3674 |  | 0.880(0.750-1.033) | 0.903(0.768-1.063) |
| Other Hispanic | 62/1454 |  | 0.701(0.533-0.923) | 0.697(0.528-0.920) |
| Other Race | 41/1378 |  | 0.857(0.620-1.184) | 0.826(0.592-1.152) |
| Marital status |  |  |  |  |
| Single | 126/4414 |  | 1 (ref) | 1 (ref) |
| Married | 854/10228 |  | 0.645(0.520-0.800)  - | 0.640(0.515-0.795) |
| Separated/divorced/widowed | 605/3643 |  | 0.845(0.682-1.070)  - | 0.837(0.667-1.050) |
| Education level |  |  |  |  |
| Primary school | 388/2156 |  | 1 (ref) | 1 (ref) |
| Junior high school | 276/2710 |  | 0.952(0/802-1.129) | 0.938(0.790-1.114) |
| Senior high school | 384/3993 |  | 0.869(0.738-1.023) | 0.872(0.740-1.027) |
| College and above | 543/8639 |  | 0.719(0.617-0.839) | 0.724(0.621-0.845) |
| Smoking status |  |  |  |  |
| Never smoker | 663/9623 |  | 1 (ref) | 1 (ref) |
| Former smoker | 598/4345 |  | 1.095(0.973-1.233) | 1.084(0.962-1.222) |
| Current smoker | 290/3157 |  | 2.014(1.729-2.346) | 2.026(1.737-2.362) |
| Hypertension |  |  |  |  |
| No | 770/13015 |  | - | 1 (ref) |
| Yes | 846/5924 |  | - | 1.038(0.929-1.161) |
| Diabetes |  |  |  |  |
| No | 1286/17139 |  | - | 1 (ref) |
| Yes | 330/1889 |  | - | 1.361(1.188-1.559) |
| Cardiovascular Disease |  |  |  |  |
| No | 1215/16129 |  | - | 1 (ref) |
| Yes | 366/1338 |  | - | 1.446(1.270-1.646) |
| Cancer |  |  |  |  |
| No | 1287/16027 |  | - | 1 (ref) |
| Yes | 309/1484 |  | - | 1.152(1.006-1.318) |

Abbreviations: CIs: confidence intervals; HR: hazard ratio; LDL-C: low-density lipoprotein cholesterol.

Model 1^a^: Age-adjusted model, adjusted for age (continuous).

Model 2^b^: Crude-adjusted model, adjusted for age (continuous), sex, race, marital status, education level, smoking status, BMI (continuous).

Model 3^c^: Fully-adjusted model, adjusted for age (continuous), sex, race, marital status, education level, smoking status, BMI (continuous), hypertension, diabetes, cardiovascular disease, cancer.

**Table S2.** Univariate analysis of LDL-C level and covariates associated with cardiovascular mortality in the NHANES Study, 1999-2014.

| Characteristics | Events/numbers | HR (95% CI) | *P* |
| --- | --- | --- | --- |
| LDL-C (mg/dL) |  |  |  |
| <70 | 25/1557 | 1.669(1.082-2.668) | 0.021 |
| 70-99 | 72/5246 | 1.229(0.932-1.809) | 0.122 |
| 100-129 | 69/6212 | 1 (ref) |  |
| 130-159 | 50/3996 | 0.980(0.666-1.442) | 0.917 |
| ≥160 | 34/2023 | 1.525(1.042-2.231) | 0.030 |
| Sex |  |  |  |
| Men | 158/9045 | 1 (ref) |  |
| Women | 92/9989 | 0.509(0.394-0.659) | <0.001 |
| Race |  |  |  |
| Non-Hispanic White | 150/8656 | 1(ref) |  |
| Non-Hispanic Black | 45/3872 | 0.693(0.497-0.967) | 0.031 |
| Mexican American | 43/3674 | 0.580(0.413-0.815) | 0.002 |
| Other Hispanic | 9/1454 | 0.475(0.242-0.931) | 0.030 |
| Other Race | 3/1378 | 0.214(0.068-0.670) | 0.008 |
| Marital status |  |  |  |
| Single | 21/4414 | 1 (ref) |  |
| Married | 127/10228 | 2.519(1.587-3.997) | <0.001 |
| Separated/divorced/widowed | 100/3643 | 6.509(4.065-10.422) | <0.001 |
| Education level |  |  |  |
| Primary school | 60/2156 | 1 (ref) |  |
| Junior high school | 46/2710 | 0.634(0.432-0.931) | 0.020 |
| Senior high school | 66/3993 | 0.625(0.440-0.886) | 0.008 |
| College and above | 77/8639 | 0.358(0.255-0.501) | <0.001 |
| Smoking status |  |  |  |
| Never smoker | 94/9623 | 1 (ref) |  |
| Former smoker | 103/4345 | 2.350(1.776-3.107) | <0.001 |
| Current smoker | 43/3157 | 1.317(0.918-1.890) | 0.134 |
| Hypertension |  |  |  |
| No | 113/13015 | 1 (ref) |  |
| Yes | 137/5924 | 3.197(2.491-4.104) | <0.001 |
| Diabetes |  |  |  |
| No | 181/17139 | 1 (ref) |  |
| Yes | 69/1889 | 4.290(3.248-5.667) | <0.001 |
| Cancer |  |  |  |
| No | 207/16027 | 1 (ref) |  |
| Yes | 43/1484 | 2.643(1.902-3.672) | <0.001 |

Abbreviations: LDL-C: low-density lipoprotein cholesterol; HR: hazard ratio; CIs: confidence intervals.

| Characteristics | Events/numbers | Multivariate HR (95% CI) | | |
| --- | --- | --- | --- | --- |
|  |  | Age-adjusted model | Crude-adjusted model | Fully-adjusted model |
| LDL-C |  |  |  |  |
| <70 | 25/1557 | 1.845(1.174-2.902) | 1.722(1.067-2.779) | 1.468(0.902-2.391) |
| 70-99 | 72/5246 | 1.542(1.107-2.149) | 1.451(1.024-2.057) | 1.342(0.945-1.908) |
| 100-129 | 69/6212 | 1(ref) | 1(ref) | 1(ref) |
| 130-159 | 50/3996 | 0.872(0.592-1.283) | 0.904(0.605-1.350) | 0.932(0.624-1.394) |
| >160 | 34/2023 | 1.307(0.893-1.913) | 1.391(0.935-2.071) | 1.447(0.971-2.156) |
| Sex |  |  |  |  |
| Men | 158/9045 | - | 1(ref) | 1(ref) |
| Women | 92/9989 | - | 0.407 (0.302-0.550) | 0.411(0.304-0.555) |
| Race |  |  |  |  |
| Non-Hispanic White | 150/8656 | - | 1(ref) | 1(ref) |
| Non-Hispanic Black | 45/3872 | - | 1.100(0.763-1.585) | 1.023(0.708-1.480) |
| Mexican American | 43/3674 | - | 0.885(0.586-1.336) | 0.798(0.527-1.210) |
| Other Hispanic | 9/1454 | - | 0.648(0.313-1.342) | 0.600(0.290-1.242) |
| Other Race | 3/1378 | - | 0.474(0.150-1.495) | 0.415(0.131-1.315) |
| Marital status |  |  |  |  |
| Single | 21/4414 | - | 1(ref) | 1(ref) |
| Married | 127/10228 | - | 0.448(0.271-0.741) | 0.439(0.266-0.725) |
| Separated/divorced/widowed | 100/3643 | - | 0.697(0.412-1.179) | 0.685(0.405-1.158) |
| Education level |  |  |  |  |
| Primary school | 60/2156 | - | 1(ref) | 1(ref) |
| Junior high school | 46/2710 | - | 1.008(0.657-1.546) | 1.009(0.658-1.546) |
| Senior high school | 66/3993 | - | 0.968(0.646-1.451) | 0.986(0.658-1.477) |
| College and above | 77/8639 | - | 0.692(0.468-1.023) | 0.704(0.476-1.040) |
| Smoking status |  |  |  |  |
| Never smoker | 94/9623 | - | 1(ref) | 1(ref) |
| Former smoker | 103/4345 | - | 1.217(0.902-1.642) | 1.233(0.913-1.664) |
| Current smoker | 43/3157 | - | 2.123(1.428-3.156) | 2.162(1.455-3.210) |
| Hypertension |  |  |  |  |
| No | 113/13015 | - | - | 1(ref) |
| Yes | 137/5924 | - | - | 1.009(0.762-1.337) |
| Diabetes |  |  |  |  |
| No | 181/17139 | - | - | 1(ref) |
| Yes | 69/1889 | - | - | 2.011(1.471-2.750) |
| Cancer |  |  |  |  |
| No | 207/16027 | - | - | 1(ref) |
| Yes | 43/1484 | - | - | 0.893(0.625-1.275) |

**Table S3**. Multivariate analysis of LDL-C levels and covariates associated with cardiovascular mortality in the NHANES Study, 1999–2014.

Abbreviations: CI: confidence intervals; HR: hazard ratio; LDL-C: low-density lipoprotein cholesterol.

Model 1^a^: Age-adjusted model, adjusted for age(continuous).

Model 2^b^: Crude-adjusted model, adjusted for age(continuous), sex, race, marital status, education level, smoking status, BMI (continuous).

Model 3^c^: Fully-adjusted model, adjusted for age(continuous), sex, race, marital status, education level, smoking status, BMI (continuous), hypertension, diabetes, cancer.

**Table S4.** Stratified analysis of the relationship between LDL-C levels and cardiovascular mortality in the NHANES Study, 1999–2014.

| Characteristics | LDL-C(mg/dL) | Numbers of Events | HR (95% CI) | *P*_value_ |
| --- | --- | --- | --- | --- |
| Sex |  |  |  |  |
| Men | <70 | 19 | 1.761(0.991-3.130) | 0.054 |
|  | 70-99 | 52 | 1.644(1.068-2.532) | 0.024 |
|  | 100-129 | 43 | 1(ref) |  |
|  | 130-159 | 27 | 0.864(0.524-1.491) | 0.643 |
|  | >160 | 17 | 1.463(0.857-2.498) | 0.163 |
| Women | <70 | 6 | 1.037(0.393-2.738) | 0.942 |
|  | 70-99 | 20 | 0.911(0.484-1.716) | 0.773 |
|  | 100-129 | 26 | 1(ref) |  |
|  | 130-159 | 23 | 1.030(0.547-1.942) | 0.927 |
|  | >160 | 13 | 1.322(0.723-2.453) | 0.358 |
| Race |  |  |  |  |
| Non-Hispanic White | <70 | 14 | 1.547(0.838-2.857) | 0.163 |
|  | 70-99 | 45 | 1.356(0.865-2.128) | 0.185 |
|  | 100-129 | 42 | 1(ref) |  |
|  | 130-159 | 29 | 0.950(0.571-1.579) | 0.842 |
|  | >160 | 20 | 1.353(0.800-2.287) | 0.260 |
| Non-Hispanic Black | <70 | 6 | 1.914(0.648-5.658) | 0.240 |
|  | 70-99 | 12 | 1.348(0.570-3.185) | 0.496 |
|  | 100-129 | 11 | 1(ref) |  |
|  | 130-159 | 9 | 1.018(0.368-2.813) | 0.973 |
|  | >160 | 7 | 1.592(0.641-3.951) | 0.316 |
| Mexican American | <70 | 4 | 0.999(0.217- 4.597) | 0.999 |
|  | 70-99 | 8 | 1.068 (0.411-2.778) | 0.892 |
|  | 100-129 | 13 | 1(ref) |  |
|  | 130-159 | 12 | 1.063(0.423-2.668) | 0.897 |
|  | >160 | 6 | 1.880(0.725-4.877) | 0.194 |
| Other Hispanic | <70 | 1 | 12.473(0.337- 462.302) | 0.171 |
|  | 70-99 | 5 | - | 0.060 |
|  | 100-129 | 2 | 1(ref) |  |
|  | 130-159 | 0 | - | 0.981 |
|  | >160 | 1 | 5.304(0.066-427.332) | 0.456 |
| Other Race | <70 | 0 | - | 0.862 |
|  | 70-99 | 2 | - | 0.756 |
|  | 100-129 | 1 | 1(ref) |  |
|  | 130-159 | 0 | - | 0.841 |
|  | >160 | 0 | - | 0.821 |
| Marital status |  |  |  |  |
| Single | <70 | 3 | 1.859(0.390-8.853) | 0.436 |
|  | 70-99 | 5 | 0.231(0.046-1.165) | 0.076 |
|  | 100-129 | 8 | 1(ref) |  |
|  | 130-159 | 2 | 0.640(0.132- 3.107) | 0.580 |
|  | >160 | 3 | 0.982(0.241- 3.996) | 0.980 |
| Married | <70 | 15 | 2.077(1.096-3.938) | 0.025 |
|  | 70-99 | 35 | 1.647(0.993-2.734) | 0.053 |
|  | 100-129 | 31 | 1(ref) |  |
|  | 130-159 | 28 | 1.081(0.614-1.901) | 0.787 |
|  | >160 | 18 | 2.154(1.223-3.794) | 0.008 |
| Separated/divorced/widowed | <70 | 7 | 0.748(0.283-1.980) | 0.559 |
|  | 70-99 | 32 | 1.373(0.802-2.352) | 0.248 |
|  | 100-129 | 30 | 1(ref) |  |
|  | 130-159 | 19 | 0.866(0.462-1.626) | 0.655 |
|  | >160 | 12 | 0.995(0.528-1.873) | 0.988 |
| Education level |  |  |  |  |
| Primary school | <70 | 8 | 2.319(0.848-6.341) | 0.101 |
|  | 70-99 | 21 | 2.144(1.024-4.492) | 0.043 |
|  | 100-129 | 13 | 1(ref) |  |
|  | 130-159 | 8 | 0.640(0.224-1.828) | 0.405 |
|  | >160 | 10 | 1.837(0.798-4.227) | 0.153 |
| Junior high school | <70 | 2 | 0.630(0.131-3.020) | 0.564 |
|  | 70-99 | 14 | 1.863(0.787-4.407) | 0.157 |
|  | 100-129 | 12 | 1(ref) |  |
|  | 130-159 | 12 | 1.670(0.685-4.072) | 0.259 |
|  | >160 | 6 | 1.837(0.688-4.904) | 0.225 |
| Senior high school | <70 | 3 | 0.732(0.212-2.529) | 0.621 |
|  | 70-99 | 19 | 1.057(0.517-2.161) | 0.879 |
|  | 100-129 | 19 | 1(ref) |  |
|  | 130-159 | 16 | 1.134(0.532-2.418) | 0.745 |
|  | >160 | 9 | 1.912(0.920-3.973) | 0.082 |
| College and above | <70 | 12 | 2.092(1.007-4.349) | 0.048 |
|  | 70-99 | 18 | 0.945(0.500-1.784) | 0.862 |
|  | 100-129 | 25 | 1(ref) |  |
|  | 130-159 | 13 | 0.639(0.319-1.279) | 0.206 |
|  | >160 | 9 | 0.829(0.382-1.798) | 0.635 |
| Smoking status |  |  |  |  |
| Never smoker | <70 | 4 | 0.407(0.120-1.382) | 0.150 |
|  | 70-99 | 24 | 1.121(0.627-2.002) | 0.700 |
|  | 100-129 | 25 | 1(ref) |  |
|  | 130-159 | 25 | 1.099(0.597-2.021) | 0.762 |
|  | >160 | 16 | 1.637(0.883-3.036) | 0.118 |
| Former smoker | <70 | 15 | 2.262(1.181-4.335) | 0.014 |
|  | 70-99 | 27 | 1.300(0.750-2.256) | 0.350 |
|  | 100-129 | 29 | 1(ref) |  |
|  | 130-159 | 18 | 0.830(0.435-1.584) | 0.573 |
|  | >160 | 14 | 1.736(0.949-3.175) | 0.073 |
| Current smoker | <70 | 5 | 2.066(0.680-6.273) | 0.201 |
|  | 70-99 | 15 | 2.353(1.052-5.259) | 0.037 |
|  | 100-129 | 13 | 1(ref) |  |
|  | 130-159 | 6 | 0.791(0.290-2.161) | 0.648 |
|  | >160 | 4 | 0.614(0.194-1.948) | 0.408 |
| Hypertension |  |  |  |  |
| No | <70 | 8 | 1.588(0.694-3.635) | 0.274 |
|  | 70-99 | 24 | 1.134(0.648-1.984) | 0.660 |
|  | 100-129 | 32 | 1(ref) |  |
|  | 130-159 | 27 | 0.901(0.512-1.588) | 0.719 |
|  | >160 | 22 | 1.557(0.911-2.661) | 0.105 |
| Yes | <70 | 17 | 1.435(0.776-2.652) | 0.250 |
|  | 70-99 | 48 | 1.488(0.939-2.359) | 0.091 |
|  | 100-129 | 37 | 1(ref) |  |
|  | 130-159 | 23 | 0.952(0.536-1.692) | 0.867 |
|  | >160 | 12 | 1.162(0.626-2.158) | 0.634 |
| Diabetes |  |  |  |  |
| No | <70 | 15 | 2.007(1.094-3.681) | 0.024 |
|  | 70-99 | 51 | 1.775(1.171-2.692) | 0.007 |
|  | 100-129 | 48 | 1(ref) |  |
|  | 130-159 | 39 | 1.092(0.691-1.726) | 0.706 |
|  | >160 | 28 | 1.512(0.945-2.420) | 0.085 |
| Yes | <70 | 10 | 1.034(0.458-2.334) | 0.935 |
|  | 70-99 | 21 | 0.776(0.401-1.499) | 0.450 |
|  | 100-129 | 21 | 1(ref) |  |
|  | 130-159 | 11 | 0.626(0.251-1.563) | 0.316 |
|  | >160 | 6 | 1.592(0.728-3.482) | 0.244 |
| Cancer |  |  |  |  |
| No | <70 | 19 | 1.273(0.720-2.248) | 0.406 |
|  | 70-99 | 54 | 1.235(0.835-1.827) | 0.290 |
|  | 100-129 | 59 | 1(ref) |  |
|  | 130-159 | 46 | 0.968(0.630-1.488) | 0.883 |
|  | >160 | 29 | 1.447(0.943-2.222) | 0.091 |
| Yes | <70 | 6 | 2.126(0.765-5.911) | 0.148 |
|  | 70-99 | 18 | 1.844(0.806-4.223) | 0.147 |
|  | 100-129 | 10 | 1(ref) |  |
|  | 130-159 | 4 | 0.703(0.217-2.280) | 0.557 |
|  | >160 | 5 | 1.322(0.442-3.954) | 0.617 |

Abbreviations: CI: confidence intervals; HR: hazard ratio; LDL-C: low-density lipoprotein cholesterol.

Adjusted for age(continuous), sex, race, marital status, education level, smoking status, BMI (continuous), hypertension, diabetes, cancer.

**Figure Legends**

**Figure 1.** Multivariate analysis of low-density lipoprotein cholesterol (LDL-C) level and covariates associated with cardiovascular mortality in the NHANES Study, 1999-2014

A: Age-adjusted model (model 1), adjusted for age (continuous).

B: Crude-adjusted model (model 2), adjusted for age (continuous), sex, race, marital status, education level, smoking status, BMI (continuous).

C: Fully-adjusted model (model 3), adjusted for age (continuous), sex, race, marital status, education level, smoking status, BMI (continuous), hypertension, diabetes, cancer.

**Figure 2.** Spline plot of low-density lipoprotein cholesterol (LDL-C) level and cardiovascular mortality rate

The adjusted odd ratios and 95% confidence intervals (CIs) were calculated with logistic regression models after adjusting for age (continuous), sex, race, marital status, education level, smoking status, BMI (continuous), hypertension, diabetes, cancer.


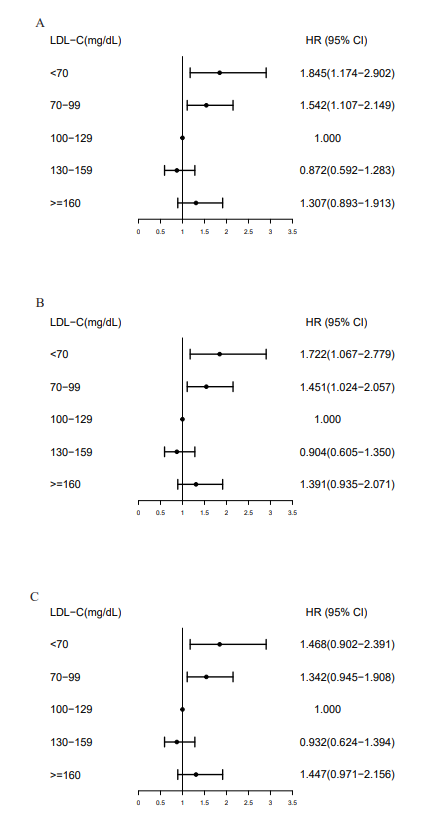


**Figure 1**


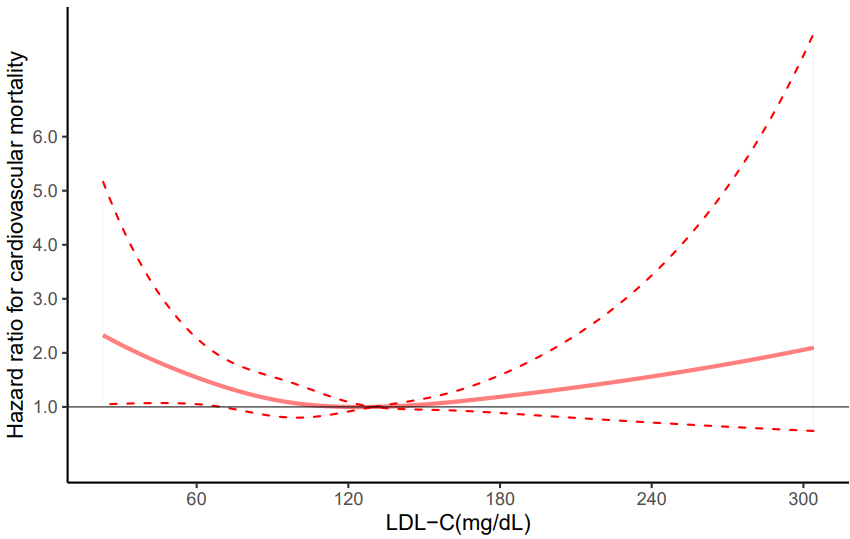


**Figure 2**
